# Filling Teeth after the Pulp-Cavity Has Become Exposed

**Published:** 1850-10

**Authors:** 


### ARTICLE III.

### *Filling Teeth after the Pulp-Cavity has become Exposed.*

The propriety of removing the dental pulp from any of the teeth farther back in the mouth than the cuspidati, has long been a mooted point with many of the most skilful in the profession of dental surgery. Some have asserted, that wherever the cavity was sufficiently large to expose the vessels, it was best at once to remove the tooth, as it never could be effectually saved and rendered serviceable. Others have contended that the tooth might be made as good as ever, by capping or otherwise protecting the uncovered nerve from pressure and the action of external agents. Others, again, have attempted the extirpation of the nerve and blood-vessels, and filling the entire length of the fang with gold.

If we accept the first as the true mode of procedure, we shall probably sacrifice a great many valuable teeth, and teeth that might with skill have been saved and made useful for many years. It is possible that most practicing dentists of *this* day make *some* attempt to save bicuspids and molars, particularly where, from the loss of many other teeth, they are much needed for mastication. The question then becomes one of vital importance, which is the best method? which is the truth?

Perhaps one of the best ways of arriving at the truth, would be for each one to "give in his experience," for, after all our theorising, experience is the great teacher. I will endeavor, in as brief and concise a manner as possible, to give the results, as far as known, of my own operations on teeth thus situated, in the past sixteen years. In 1831 I had the pleasure of witnessing the extirpation of the pulp, and the filling of the fang to the apex of a central incisor, in the mouth of a lady, by that accomplished dentist, Dr. Edward Hudson, of Philadelphia, and have had the opportunity of seeing this tooth frequently since; it became somewhat discolored, but not sufficiently to render its presence offensive, and still remains a serviceable tooth—the filling being as sound as when first put in. The success attending this operation induced me to attempt, in 1833, the filling of four incisors for a gentleman, all of which I filled in the fangs as far up as I could succeed in passing a small probe. These teeth I saw in 1846—thirteen years after—and they looked well, had never had any abscess, or any irritation in their sockets. The first attempt I ever made to save a tooth by capping a nerve, was in 1836: a large superior molarin the approximal surface, the bicuspid having been removed, and thereby affording an excellent opportunity for a perfect operation. Upon removing the caries, the nerve became apparent, and a slight redness was seen, though there was no bleeding of the vessels. I made a concave cap of gold, sufficiently strong to resist any pressure I could exert with my instrument in filling, and placed it very carefully over the part, taking great care that the edges should have a firm bearing on the sound surrounding bone, and then filled the cavity all around and over it; and after polishing the filling, and finding that cold water thrown on the tooth and filling from a syringe produced no sensation, concluded the operation was perfectly satisfactory, and the tooth effectually preserved. At the end of one year, the patient called on me to have other teeth examined, and said this tooth had done very well; that though occasionally it gave him a thrill, it never amounted to pain. In six months more I again saw him, and then found that an abscess was forming, and upon removing the filling and cap, the pus gushed from the opening. This relieved it for the time, but in a few days, the tooth becoming more painful, he begged to have it removed, which was accordingly done.

In 1837, a lady called on me, complaining of a roughness between her central incisors, though she had no idea there was any amount of decay—supposed a slight filing would remove it. Upon making a separation with a file, considerable caries was found in one of the teeth, upon removing which, the pulp was almost reached, but not wounded, so as to produce pain. Being very unwilling, as the teeth were very beautiful both in shape and color, to destroy the nerve, and thereby involve the probability of changing the hue of the tooth, I concluded to fill over it, and watch it carefully from time to time, so that the first approach of danger might be noted, and the nerve extirpated and fang filled if it should prove necessary. One year after, I received a note, requesting me to advise what should be done with this tooth, as the lady had been suffering some days. Upon calling to see her, I found that she had felt pain in it for several months, and it had become almost black.

I immediately removed the filling, which gave relief from the pain, and after some treatment, filled the fang and crown, but the color of the tooth I never could restore. The whole bony structure had become so much discolored that it was impossible to restore it. This, with some variations of condition and appearance, modified by the character of the teeth and temperament of the patient, has been the result sooner or later, in nearly all my attempts at filling teeth over exposed nerves. There remains for consideration now, only the last mentioned method of treatment, to wit: the operation of extirpating the diseased or exposed tissue and filling the cavity. My experience convinces me that wherever the vascular and nervous branches distributed in the bone are ruptured and their connexion with the bone severed, that the pulp, if left remaining as in the operation of capping, dies sooner or later, and when it dies in this way, it involves the early death and loss of the tooth. The pulp becomes a center of irritation, inflammation extends to the external membrane, and the tooth perishes by the loss of its only remaining supply of vitality. This is not so likely to happen when the nerve and vessels are skilfully taken out and the fangs are filled with gold. When this operation is successful, the circulation in the periostial membrane remains as perfect as ever, and the body of the tooth naturally supplied by this route, is still healthfully supported; and it is moreover clear by all the analogies of the circulation, that even the most interior parts of the structure must derive an adequate supply of blood through the anastomosing radicals of excised branches left by the operation of extirpation; and so the prolonged *life* of the tooth, which experience proves, is maintained and accounted for. Of course the utility of this operation will depend upon the perfectness with which it is performed.

It is often very difficult to reach all the fangs of a molar tooth in the upper jaw, and great expertness is necessary to fill them so as to leave no room above the filling for the accumulation of pus. Since the year 1840, I have filled a great many teeth in the fangs, and as far as my note book furnishes evidence of the results, eighty out of one hundred cases have been successful, and I am inclined to the belief, that most of the failures have been owing to my imperfect performance of the operation, rather than a mistake in the propriety of the operation itself, for in those which I have extracted some months afterwards I have found almost in every case, either, that the vessels were not entirely extirpated, or that my filling had not reached quite so far as it should.

From the result of my own operations, therefore, I have arrived at this conclusion, which I must hold until further experience proves its fallacy. That a tooth, the nerve of which is exposed, and the circulation of the blood-vessels cut off, must and will, sooner or later, become an ulcerated and diseased tooth, demanding removal, and perhaps involving the alveolus in its disease ; and, that by thoroughly extirpating the pulp and filling the cavity, eighty per cent. may be saved effectually.

E. T.

---

